# Sentiment Analysis of Persian Movie Reviews Using Deep Learning

**DOI:** 10.3390/e23050596

**Published:** 2021-05-12

**Authors:** Kia Dashtipour, Mandar Gogate, Ahsan Adeel, Hadi Larijani, Amir Hussain

**Affiliations:** 1 Department of Computing Science and Mathematics, University of Stirling, Stirling FK9 4LA, UK; 2 School of Computing, Edinburgh Napier University, Edinburgh EH11 4BN, UK; m.gogate@napier.ac.uk (M.G.); Amir.Hussain@napier.ac.uk (A.H.); 3 School of Mathematics and Computer Science, University of Wolverhampton, Wolverhampton WV1 1LY, UK; aadeel200@gmail.com; 4 School of Computing, Engineering and Built Environment, Glasgow Caledonian University, Glasgow G4 0BA, UK; H.Larijani@gcu.ac.uk

**Keywords:** sentiment analysis, deep learning, CNN, LSTM, classification

## Abstract

Sentiment analysis aims to automatically classify the subject’s sentiment (e.g., positive, negative, or neutral) towards a particular aspect such as a topic, product, movie, news, etc. Deep learning has recently emerged as a powerful machine learning technique to tackle the growing demand for accurate sentiment analysis. However, the majority of research efforts are devoted to English-language only, while information of great importance is also available in other languages. This paper presents a novel, context-aware, deep-learning-driven, Persian sentiment analysis approach. Specifically, the proposed deep-learning-driven automated feature-engineering approach classifies Persian movie reviews as having positive or negative sentiments. Two deep learning algorithms, convolutional neural networks (CNN) and long-short-term memory (LSTM), are applied and compared with our previously proposed manual-feature-engineering-driven, SVM-based approach. Simulation results demonstrate that LSTM obtained a better performance as compared to multilayer perceptron (MLP), autoencoder, support vector machine (SVM), logistic regression and CNN algorithms.

## 1. Introduction

Sentiment analysis is a method to automatically classify large amounts of text into positive or negative sentiments [1,2,3,4]. With the explosive growth of social media, organizations and companies have started to use big online data for their product enhancement and proactive decision making. In recent years, social media, forums, blogs, and other forms of online communication tools have radically affected everyday life, especially how people express their opinions. The extraction of useful information (for example, people’s opinions about company brands) from the huge amount of unstructured data is important for most companies and organizations [5,6,7,8,9]. The application of sentiment analysis is not limited to product or movie reviews. Researchers have also applied it to areas such as news, politics, sport, etc. For example, in online political debates, sentiment analysis can be used to identify people’s opinions about a certain candidate or political party [10,11,12,13,14,15,16]. However, sentiment analysis has been widely used for the English language using traditional and advanced machine learning techniques, and limited research has been conducted to develop models for the Persian language [17,18,19,20].

In the literature, Sentiment analysis has been performed at both the document and sentence level. The document-level sentiment analysis has been used to classify the sentiments expressed in the document (positive or negative), whereas, at the sentence level, the models have been used to identify the sentiments expressed only in the sentence under analysis [21,22,23,24,25,26,27]. For sentiment analysis, there are two widely used approaches: (1) Lexicon-based approach that uses lexicons (a dictionary of words and corresponding polarities) to assign polarity; (2) Machine learning approach which requires a large labelled dataset with manual annotation [28,29,30,31,32]. Recently, deep-machine-learning-based automated feature engineering and classification has been shown to outperform state-of-the-art, manual-feature-engineering-based shallow classification. However, both deep and shallow machine learning algorithms have widely been applied to English Corpora, with little work being carried out to develop deep learning models for Persian sentiment analysis [33,34,35,36,37,38,39,40,41,42].

Persian is the official language of Iran and Afghanistan, with more than one hundred million speakers. A large amount of unstructured text is available online, including newspapers, books, web pages, movie reviews, etc. [43,44,45]. In this study, a novel corpus for Persian sentiment analysis is developed and evaluated using both shallow and deep machine learning algorithms. For shallow learning, logistic regression, support vector machine (SVM), and multilayer perceptron (MLP) classifiers are used. For deep learning, 1D convolutional neural network (CNN), 2D-CNN, stacked long short-term memory (LSTM), and Bidirectional LSTM algorithms have been utilized.

To the best of our knowledge, this is the first work on exploiting deep-learning-based automated feature engineering for Persian sentiment analysis. In addition, the *fastText* word embedding is used to obtain a vector representation of Persian words. The contextual features are extracted using deep learning algorithms and their polarity detection performances are compared with traditional shallow classifiers.

In summary, the paper reports two major contributions, outlined below:

1. We proposed novel architectures for deep learning classifiers such as convolutional neural network (CNN) and long short-term memory (LSTM) to identify the polarity of the Persian text;
2. An ablation study of our proposed deep learning reveals the importance of individual layers in the context of the complete framework.

The rest of the paper is organized as follows: In Section 2, related work on sentiment analysis for English, Persian, and other languages is presented. Section 3 presents our proposed novel approach for Persian sentiment analysis. In Section 4, experimental results are presented. Finally, Section 5 concludes this work with suggestions for future work.

## 2. Related Work

### 2.1. English Language

Recently, Pitsilis et al. [46] proposed a framework to discern hateful content in social media using a Recurrent Neural Network (RNN) classifier. The authors collected data from Twitter and fed the extracted features into an RNN classifier. The results demonstrated the effectiveness of RNN (achieving up to 95.33%) as compared to a state-of-the-art machine learning algorithm such as SVM (75.22%). Chen et al. [47] proposed a novel framework to improve sentence-level sentiment analysis by employing Long-Short-Term Memory with a conditional random field layer (BiLSTM-CRF). The comparative simulation results with benchmark datasets showed that their proposed framework improved the overall accuracy of sentence-level sentiment analysis. Hassan et al. [48] proposed an approach to detect polarity using CNN and LSTM using pre-trained word vectors of IMDB movie reviews. The CNN classifier developed in this approach consisted of two convolutional layers and two pooling layers. The experimental results showed that the combined CNN and LSTM model achieved up to 88.3% accuracy and outperformed CNN (87%) and LSTM (81.8%) models. Similarly, Shen et al. [49] proposed a novel approach to combine CNN and bidirectional Long-Short-Term Memory (BLSTM) to detect polarity for movie reviews. The combined CNN-LSTM model outperformed CNN and LSTM classifiers by achieving an accuracy of up to 89.7% as compared to 83.9% and 78.4%, respectively.

Nguyen et al. [50] proposed a novel method to detect polarity in news articles using a deep learning classifier. The authors used different websites to collect more than one million news articles and fed the preprocessed embedded vectors into CNN, LSTM and convolutional LSTM (CLSTM). The comparative simulation results showed that the CLSTM model outperformed CNN and LSTM classifiers by achieving an accuracy of up to 96.52% as compared to the 92.3% and 91.19% accuracy achieved by CNN and LSTM, respectively. Liao et al. [51] proposed a framework to understand users’ satisfaction with a product using a deep learning classifier. The authors used the Twitter API to collect the tweets. The CNN classifier developed in this framework contained one convolutional layer and one pooling layer. Their experimental results showed that CNN outperformed SVM and Naive Bayes classifiers by achieving the accuracy up to 95% as compared to the 70% and 62.25% accuracy achieved by SVM and Naive Bayes, respectively. Zhao et al. [52] proposed a model to detect sentiment in product reviews using a deep learning classifier. The words are first converted into an embedding vector representation, and then a deep learning classifier is used to classify sentiments. The experimental results showed the effectiveness of their proposed model, where CNN achieved up to 87% accuracy as compared to LSTM (82%). Ouyang et al. [53] proposed a framework to detect polarity in English movie reviews using a deep learning classifier. The movie reviews were collected from rottentomatoes.com. The dataset consisted of five labels: positive, somewhat positive, neural, somewhat negative and negative. The experimental results showed that the RNN achieved an accuracy of up to 78.34% as compared to SVM (62%).

### 2.2. Persian Language

Basiri et al. [54] proposed a lexicon for Persian that consisted of 150,000 sentences along with labels indicating the polarity of the sentences. The authors used a Naive Bayes classifier to evaluate the performance of their proposed approach and achieved 91% accuracy. Ebrahimi et al. [55] proposed a supervised method to develop a sentiment lexicon for the Persian language by extracting features in movie reviews. The authors used an SVM classifier to evaluate the performance of the approach and achieved 80% accuracy. Saraee et al. [56] proposed a novel approach to detect polarity in Persian movie reviews using n-gram features. Their proposed approach consisted of stemming and feature selection. The authors used a Naive Bayes classifier to evaluate the performance and achieved 82.26% accuracy.

Amiri et al. [57] developed a lexicon to detect polarity for multi-domain product and movie reviews in Persian. The authors collected sentences from people’s communication and then pre-processed them. The authors used an SVM classifier to evaluate the performance of the lexicon and achieved an accuracy of 69%. Vaziripour et al. [58] proposed an approach to automatically classify the sentiment in Persian tweets. The experimental results showed that SVM achieved up to 70% accuracy as compared to Naïve Bayes (56%). Sabeti et al. [59] developed a lexicon for Persian. The lexicon achieved an accuracy of 81.06% using K-nearest neighbors (KNN).

Basiri et al. [60] proposed a method to detect polarity in Persian product reviews. The experimental results showed that SVM achieved an accuracy up to 68% and outperformed Naive Bayes (62%) and MLP (60%) classifiers. Alimardani et al. [61] proposed an approach to detect polarity in Persian hotel reviews. The developed lexicon contained more than one thousand words along with their polarity. The proposed approach collected hotel reviews and extracted part-of-speech tag features. The performance analysis revealed that logistic regression outperformed the SVM classifier by achieving an accuracy up to 85.9% as compared to the 82.4% accuracy achieved by SVM.

### 2.3. Other Languages

Day et al. [62] proposed a framework to explore the impact of deep learning for sentiment analysis on Google App mobile reviews in Chinese. CNN outperformed SVM and Naive Bayes classifiers by achieving an accuracy up to 94% as compared to the 76.46% and 74.12% accuracy achieved by SVM and Naive Bayes, respectively. Valverde et al. [63] proposed a novel approach to detect polarity for Spanish product services using a deep learning classifier. The product reviews were manually collected from e-commerce websites. After the pre-processing, the word embeddings was used to obtain the vectors of the words, followed by the CNN classifier. The authors achieved 88.7% accuracy.

Baniata et al. [64] proposed a sentiment analysis method to identify the polarity for Arabic text using a deep learning classifier. The combination of CNN and LSTM classifiers, trained on Arabic reviews, achieved a high accuracy of 86.43% in comparison to CNN (66.26%) and LSTM (65.34%). Xiao et al. [65] proposed an approach to detect polarity in Chinese text. The authors used 1D and 2D CNN classifiers and achieved the highest accuracy, of 93.4%, with 2D CNN. Furthermore, the experimental result showed that the character-level approach outperformed word embedding for Chinese words.

Dahou et al. [66] proposed an approach to detect polarity for Arabic text. The authors used a web crawler to build a corpus and trained word embedding to represent words in the corpus. The presented results showed that a CNN classifier with Arabic reviews outperformed other state-of-the-art supervised learning algorithms, achieving 89.7% as compared to SVM (72.4%) and Naïve Bayes (69.5%). Le et al. [67] proposed an approach for sentiment analysis for the Indonesian language to detect the polarity in the sentences or documents. The authors used four thousand movie reviews, labelled manually. Shaung et al. [68] proposed an approach using CNN-LSTM to detect polarity in English and Chinese product reviews. Their proposed CNN-LSTM model outperformed individual CNN and LSTM classifiers and achieved 81.86% accuracy. Wehrmann et al. [69] proposed a method to detect polarity in four different languages. However, their proposed method used machine translation to translate English tweets into German, Portuguese and Spanish. The experimental results showed the effectiveness of their proposed model and achieved up to 76.2% accuracy with CNN, as compared to LSTM, which achieved 64.7% accuracy. More recently, Sankar et al. [70] proposed a novel framework, which uses training and test data from different datasets, such as IMDB and Rotten Tomatos, and applies deep learning classifiers to identify the polarity of the sentence. In other hand, Ali et al. [71] implemented a sentiment classification approach using deep learning algorithms such as LSMT and CNN and hybrid CNN and LSTM to identifty the polarity of the sentence. However, most of the approaches are not suitable to determine the polarity for Persian sentences.

## 3. Methodology

**Figure 1:**
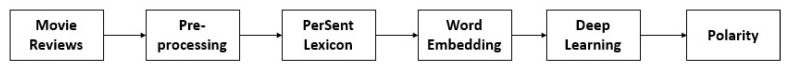
Proposed Framework.

In this section, the proposed approach for Persian movie reviews is discussed in detail. Figure 1 depicts the proposed framework, and details are presented in subsequent sections.

### 3.1. Data Pre-Processing

**Figure 2:**
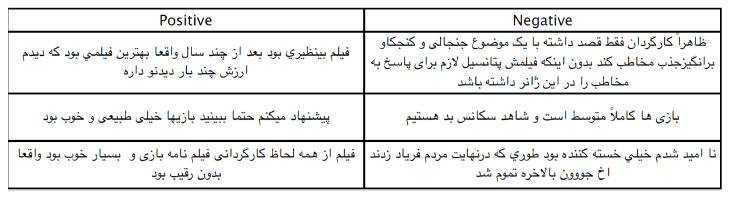
Persian Sentences Examples.

The novel dataset used in this work is collected manually from Persian movie websites such as www.caffecinema.com (accessed on 15 March 2021) and www.cinematicket.org (accessed on 15 March 2021). A subset of the dataset was used to train the neural network (60% training dataset), and the rest of the data (40%) were used to test and validate the performance of the trained neural network testing set (30%), and validation set (10%). There were two types of label in the dataset: positive and negative. The reviews were manually annotated by three native Persian speakers aged between 30 and 50 years old. The reviewers received a degree in Persian from the University of Tehran. There were 1021 positive and 989 negative reviews. After data collection, the dataset was pre-processed using tokenisation and normalisation techniques. The process of breaking a sentence into words is called tokenisation. In order to tokenise Persian sentences, the NLTK tokeniser is used. For example, “The movie is great” is tokenised into “The”, “movie”, “is”, and “great”. The normalisation technique is used to replace abbreviations and replace them with their actual meaning. In addition, normalisation is used to replace words with their actual meaning. For example, “I am happyyyyyyyy” is normalised to “I am happy”. A stemmer is used to change the words into their roots. For example, “fishing” or “fisher” is stemmed into “fish”. In order to do the stemming process, a Persian Python tool called HAZM is used. HAZM contains text cleaning, sentence word tokenizer, word lemmatizer, part-of-speech tagger, shallow parser and dependency parser [72,73,74,75,76]. Figure 2 shows examples of Persian sentences.

N-gram features are extracted from text. The n-gram size one is called unigram, size two is bigram and size three is trigram [77]. The n-gram features are extracted from Persian movie reviews. HAZM reads the text and assigns POS tag into words [78]. These tags are noun, verb, adjective, adverbs and etc. The HAZM tool is used to extract POS tag (Adjective, Adverb, Verb and Noun) from Persian movie reviews. After extracting n-grams and POS tag features, the PerSent lexicon is used to asssign polarity into features. The lexicon contains 1500 Persian words along with their part-of-speech tag (POS) and the polarity of the words [21].

**Figure 3:**
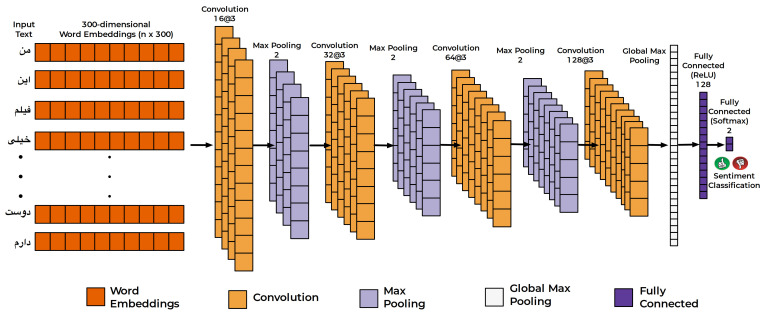
CNN Classifier.

**Word embedding:** In order to train the CNN classifier, the Persian tokenized words are converted into a three hundred dimensional vector using fastText pretrained embedding Python package. Figure 3 shows the process of word embedding with the Persian sentence example, <mn iyn fylm xyly dwst dArm>.

### 3.2. Classification

#### Convolutional Neural Network

The developed CNN classifier is shown in Figure 3. The CNN classifier consists of input, output, and hidden layers. The hidden layers constitute of convolutional, pooling, fully connected and normalization layers [33,79,80]. In experiments, the best results were obtained using an 11-layered CNN architecture, as shown in Figure 3. In the first convolutional layers, 16 feature maps with a kernel size of 2 are used. In the second convolutional layer, 32 feature maps with a kernel size 2 are used. The layers are followed by a max pooling layer of size 2. In the fourth convolutional layer, 64 feature maps with a kernel size 2 are used. The final convolutional layer is followed by a max pooling with window size 2. The feature extraction framework is followed by fully connected layers of size 5000, 500 and 2.

**Figure 4:**
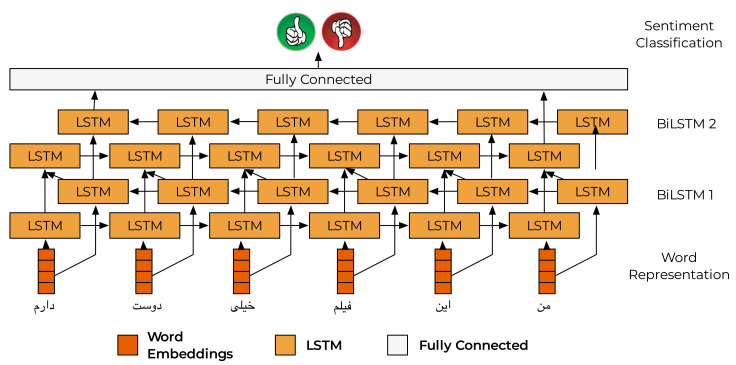
Long Term Short Memory.

**Long Short Term Memory (LSTM):** LSTM is a successful augmented recurrent neural network model which is used to learn sequential information with dependencies that LSTM can store and use to compute information for a long period of time. LSTM was originally proposed in [81] by Hochreiter et al. The developed LSTM network, presented in Figure 4, consists of an input layer, two stacked LSTM layers, and a fully connected layer. Specifically, the network consists of two bidirectional LSTM layers followed by two dropout layers, one dense layer and one activation function layer. The fastText word embedding is fed into stacked LSTM layers. The output of the second LSTM layer was then fed into the fully connected (dense) layer with two neurons. The architecture was trained using an Adam optimizer with a dropout probability of 0.2. The main reason for employing LSTM is its recurrent nature and ability to help the model with classification data. It has to be noted that previous works, such as [82,83,84,85], reveal that LSTM, due to its inherent recurrent nature, can have better model long-term dependencies and exploit temporal correlation in inputs, as compared to the MLP.

**MultiLayer perceptron (MLP):** MLPs are made up of highly interconnected processing elements called neurons, processing information by their state response and learning from examples. A neuron in an MLP is connected to several inputs with different associated weights. The output of a neuron is the summation of all connected inputs, followed by a non-linear processing unit, called a transfer function. The main objective of MLP is to transform the inputs into meaningful outputs by learning the input–output relationship, and offering viable solutions to unseen problems (a generalization capability). Therefore, the capacity to learn from examples is one of the most desirable features of neural network models. The goal of training is to learn the desired system behavior and adjust the network parameters (interconnections weights) to map (learn) the input–output relationship and minimize the cost function. The processed embedding vectors were fed into an MLP model with 1 or 2 hidden layers and 10 to 150 hidden neurons per layer.

**Autoencoder:** An autoencoder (AE) is a type of unsupervised learning algorithm, typically used for dimensionality reduction purposes. The AE standard configuration includes one input layer, one output layer and one hidden layer. It compresses the input data *x* into a lower dimension *h* through the encoding process:

(1) h=g(xw+b)

where *x*, *w*, *b* are the input vector, weight matrix, the bias vector, respectively and *g* is the activation function. Then, it attempts to reconstruct the same set of input (*x*) from the compressed representation (*h*) through the decoding process:

(2) $$\tilde{x}=g\left(hw^{T}+b\right)$$

## 4. Experimental Results

In this section, we describe the experimental setup, followed by results and discussions. To evaluate the performance of the proposed approach, movie reviews are used. For data labelling, the PerSent lexicon is used, which assigns a polarity to individual events in the dataset. The trained and test dataset is converted into vectors to train LSTM, CNN, and SVM classifiers. The n-gram features (bigram, trigram) and POS features (noun, adjective, verb and adverb) are extracted from Persian movie reviews. The extracted features are converted into bag of words and principal component analysis is used to reduce the dimensionality of data to two hundred dimensions. The extracted features are fed into an SVM and a logistic regression classifier.

The parameters of LR, SVM, LSTM, and CNN models are as follows: word embedding dimensions are 300, the number of epochs equal to 200, and batch size is 128. The ML classifiers are trained to classify the sentences into either positive or negative. In addition, it has been shown that the size of the filter has a positive impact on the final results and model achieved a better performance when the filter size was set to a smaller number such as 3.

Table 1 presents the results of SVM using various features and combination of the features. The results shows that nouns outperformed other features. For testing with LSTM and CNN, movie reviews are converted into three hundred dimensional vectors using fasttext. Table 3 presents the results of CNN and LSTM classifiers and a comparison with MLP and autoencoder. The experimental results demonstrate the effectiveness of the autoencoder as compared to MLP. To evaluate the performance of proposed approaches, precision, recall, f-Measure, and prediction accuracy are used as performance matrices

(3) $$Precision=\frac{TP}{TP+FP}$$

(4) $$Recall=\frac{TP}{TP+FN}$$

(5) $$F\_measure=2*\frac{Precision*Recall}{Precision+Recall}$$

(6) $$Accuracy=\frac{TP+TN}{TP+TN+FP+FN}$$

where TP represents, TN, FP, and FN represents true positive, true negative, false positive, and false negative, respectively.

Table 2 indicates the results for SVM and logistic regression. The experimental results shows that the noun achieved better performance as compared to other features such as adjective, adverb, verb, bigram and trigram. The experimental results demonstrate that the LR achieved better accuracy, precision, recall and f-measure as compared to SVM.

The experimental results indicated that using deep learning approaches enhances the performance of sentiment analysis in Persian dataset. The proposed model does not required any feature engineering to extract special features such as n-gram, POS and concept extraction like previous approaches. It is to be noted that the deep learning approaches are based on the pre-trained word vector representation; despite the complexity of the Persian language and the simplicity of the proposed model, there are significant improvements in the BiLSTM in terms of the F-measure and the accuracy compared to traditional machine learning classifiers.

On the other hand, deep learning classifiers achieved a better performance, such as the BiLSTM and 2D-CNN, with fastText word embedding used as features. The fastText model consists of high-quality vector representation using the semantic and syntactical information from the texts; it can also cover the out-of-vocabulary words.

The deep learning approaches are black-box; we cannot understand how the sentiment polarity assigned to sentences, for example, the polarity shifters such as negation, can change the overall polarity of the sentences. However, the Persian language consists of rhetorical and sarcastic sentences which cannot be detected by black-box deep learning approaches.

The deep learning architectures trained and validated using TensorFlow library and NVIDIA Titan X GPU. The deep learning models were trained for 200 epochs using back propagation, with the Adam optimizer minimizing the categorical cross entropy loss function.

Table 3 presents the results of SVM and LR using various features on hotel reviews. Experimental results indicate that the LR achieved better accuracy as compared with SVM. The results show that the use of adjective outperformed other features. Additionally, the adverb feature achieved lower accuracy as compared with other features.

Furthermore, Table 4 presents the results of CNN and LSTM classifiers on the hotel reviews dataset. Experimental results show the 2D-CNN achieved better accuracy as compared with other classifiers. Additionally, the stacked-BiLSTM received a lower performance as compared with other classifiers; however, it achieved better precision as compared with other classifiers.

Table 5 presents the comparison results for the proposed 1D-CNN with different layers. Experimental results show that the five-layered 1D-CNN architecture generally outperforms other layered architecture. The time to train each model is shown in the last column of Table 5 Additionally, the recall and F-measure for five-layers outperformed other architectures compared with 2-, 3-, 4-, and 6-layered models.

Table 6 presents the comparison results for the proposed 2D-CNN with different layers. Experimental results show that the five-layer CNN generally outperforms other layers. The time to train each model is shown in the last column of Table 6. Additionally, the recall and F-measure for layer 5 outperformed other layers.

Table 7 presents the comparison results for different layers of LSTM. Experimental results show that the two-layer LSTM achieved better results as compared with other layers. The time to train the model is shown in Table 7. Additionally, experimental results show that the two-layer LSTM achieved better precision, recall, f-measure as compared with other layers.

Table 8 presents the comparison results for different layers of BiLSTM. Experimental results show that the two-layer BiLSTM achieved better results as compared with the one-layer BiLSTM. Additionally, experimental results show that the two-layer BiLSTM achieved better precision, recall and f-measure as compared with the one-layer BiLSTM. Experimental results show the training time is increased as the number of layers increases.

The results demonstrate that the stacked-BiLSTM model outperformed all other methods. In addition, it is observed that CNN and LSTM classifiers can effectively detect the polarity of the movie reviews. Furthermore, they also help in detecting contextual information as compared to traditional classifiers. It is shown that deep learning approaches are more optimal for sentimental analysis due to their having less over-fitting and better generalization.

The hotel reviews corpus is used to compare how our approach performs in a new domain compared to state-of-the-art approaches, including multilingual methods. The hotel reviews were collected and, after data pre-processing, the extracted features were converted into TF-IDF and the machine learning was applied to evaluate the performance of the approach. The overall accuracy of their proposed approach is 87%. However, for their experiments, a five-fold cross-validation was used [61]. In our experiments, we used 60% for training, and the rest of the data were used for testing and to validate the performance of the trained classifiers, with 30% used for testing and 10% for the validation set.

**Figure 5:**
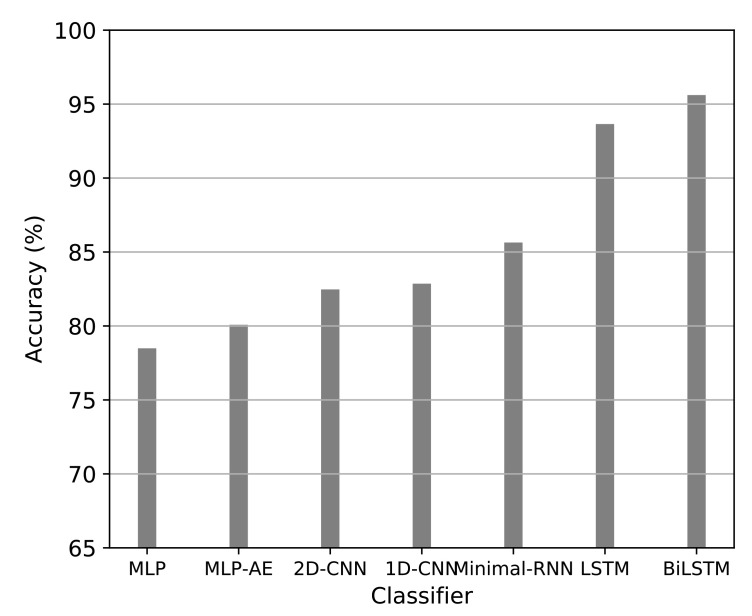
Deep Learning Classifiers’ Accuracy on Movie reviews.

Figure 5 presents the accuracy of both shallow and deep learning classifiers. It can be seen that the stacked-bidirectional-LSTM outperformed all other machine learning algorithms.

**Figure 6:**
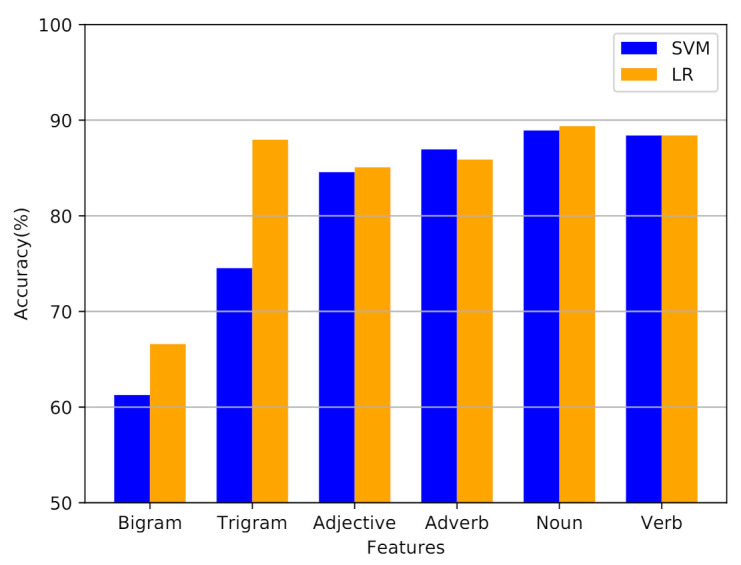
SVM vs. LR Classifier Accuracy on Movie reviews.

Figure 6 depicts the classification performance of SVM and LR using different feature types. It can be seen that the noun features help in achieving a better performance as compared to other features.

The results demonstrate that the stacked-BiLSTM model outperformed all other methods. In addition, it is observed that the CNN and LSTM classifiers can effectively detect the polarity of movie reviews. Furthermore, they also help in detecting contextual information as compared to traditional classifiers. It is shown that the deep learning approaches are more optimal for sentimental analysis due to their having less over-fitting and better generalization.

The LSTM achieved a better performance than shallow learning because, in deep learning approaches, there is no requirement for feature selection. The experimental results show that the LSTM can efficiently reflects the delays and make the input more convenient, and helps the LSTM achieve a better performance by optimizing the input form and network structure based on the clearer physical meaning. However, in order to improve the performance further, there is a need to develop an additional lexicon or grammar-rule based approach to detect sarcastic and irony sentences’ polarity. For example <ĉy bgm, nmydwnm xwb bwd yA bd bwd>, “What to say, I do not know the movie was good or bad”. The experimental results indicated that the word embedding models can identify the overall sentiment polarity of the sentiment effectively. The classification performance is better for movie reviews than casual comments. This shows the feasibility of an LSTM-based approach for Persian movie sentiment classification.

The trigram performed well as compared to the bigram, as it is more informative; for example, it consists of three word combinations, making it easier for the machine learning model to identify the overall sentiment polarity. For example, <fylm xwb bwd> “movie was great” as opposite to <fylm> “movie”. However, other studies, such as [88], showed that trigrams achieved a better performance as compared to bigrams.

## 5. Conclusions

With the rise of social media, people around the world openly share their opinions on different topics, movies, products, politics, etc. Sentiment analysis is widely used to automatically classify these sentiments as positive or negative. In the literature, much research has been conducted with English corpora but very little effort has been devoted to Persian sentiment analysis. In this study, a novel Persian architecture is developed for deep learning approaches. In order to evaluate the performance of the approach, the Persian movie reviews and hotel review dataset are used to test and validated using both shallow and deep learning algorithms. The simulation results demonstrate that deep learning outperformed state-of-the-art, shallow machine learning approaches. Specifically, the stacked-bidirectional-LSTM achieved the highest accuracy, of up to 95.61%, for the movie dataset, but not for the hotel dataset, where 2D-CNN did better (89.76%). Furthermore, we have evaluated the best performance of deep learning classifiers with different layers. Ongoing and future work aims to extend the developed corpus to multilingual product reviews. 

## Figures and Tables

**Table 1:** Deep Learning Classifiers Results on Persian Movie reviews.

Classifier	Precision	Recall	F-Measure	Accuracy (%)
Ghasemi et al. [86]	0.63	0.62	0.63	63.94
Asli et al. [87]	0.68	0.67	0.68	68.23
Amiri et al. [57]	0.69	0.68	0.69	70.01
Basiri et al. [54]	0.72	0.71	0.72	72.81
MLP	0.78	0.78	0.78	78.49
MLP-Autoencoder	0.8	0.8	0.8	80.08
2D-CNN	0.82	0.82	0.82	82.47
1D-CNN	0.84	0.83	0.83	82.86
Minimal-RNN	0.86	0.86	0.86	85.64
Stacked-LSTM	0.94	0.94	0.94	93.65
**Stacked-BiLSTM**	0.96	0.96	0.96	95.61

**Table 2:** SVM vs. Logistic Regression (LR) on Persian Movie reviews.

Feature	Precision		Recall		F-Measure		Accuracy (%)	
	**SVM**	**LR**	**SVM**	**LR**	**SVM**	**LR**	**SVM**	**LR**
Bigram	0.65	0.66	0.61	0.67	0.53	0.65	61.27	66.59
Trigram	0.81	0.88	0.75	0.88	0.71	0.88	74.53	87.96
Adjective	0.72	0.87	0.85	0.85	0.78	0.79	84.57	85.07
Adverb	0.76	0.81	0.87	0.86	0.81	0.82	86.95	85.88
**Noun**	0.79	0.91	0.89	0.89	0.84	0.85	88.93	89.37
Verb	0.78	0.78	0.88	0.88	0.83	0.83	88.4	88.4

**Table 3:** SVM vs. LR on Hotel reviews dataset.

Feature	Precision		Recall		F-Measure		Accuracy (%)	
	**SVM**	**LR**	**SVM**	**LR**	**SVM**	**LR**	**SVM**	**LR**
Bigram	0.71	0.72	0.71	0.72	0.71	0.72	71.25	72
Trigram	0.73	0.74	0.73	0.74	0.73	0.74	73.5	74
**Adjective**	0.75	0.76	0.78	0.79	0.76	0.77	76.24	77.06
Adverb	0.63	0.64	0.62	0.63	0.62	0.62	62.72	62.78
Noun	0.67	0.69	0.68	0.70	0.67	0.69	68.21	69.08
Verb	0.68	0.69	0.69	0.71	0.68	0.69	68.54	69.97

**Table 4:** Deep Learning Classifiers Results on Hotel reviews dataset.

Classifier	Precision	Recall	F-Measure	Accuracy (%)
Stacked-BiLSTM	0.53	0.91	0.67	74.49
1D-CNN	0.73	0.80	0.76	78.02
Stacked-LSTM	0.79	0.81	0.80	81.04
**2D-CNN**	0.89	0.89	0.89	89.76

**Table 5:** Comparison of 1D-CNN Layers on Persian Movie reviews.

Layer	Precision	Recall	F-Measure	Accuracy (%)	Time
2	0.72	0.73	0.72	73.55	2 m 31 s
3	0.74	0.74	0.74	74.26	3 m 12 s
4	0.81	0.79	0.79	79.01	3 m 44 s
**5**	0.84	0.83	0.83	82.86	4 m 22 s
6	0.78	0.76	0.76	78.42	5 m 24 s

**Table 6:** Comparison of 2D-CNN Layers on Persian Movie reviews.

Layer	Precision	Recall	F-Measure	Accuracy (%)	Time
2	0.72	0.70	0.70	74.05	2 m 21 s
3	073	0.71	0.71	74.22	3 m 23 s
4	078	0.78	0.78	78.26	5 m 5 s
**5**	0.82	0.82	0.82	82.47	6 m 33 s
6	0.75	0.76	0.75	76.51	8 m 25 s

**Table 7:** Comparison of LSTM Layers on Persian Movie reviews.

Layer	Precision	Recall	F-Measure	Accuracy (%)	Time
**2**	0.94	0.94	0.94	93.65	6 m 49 s
3	0.84	0.83	0.83	84.26	7 m 28 s

**Table 8:** Comparison of BiLSTM Layers on Persian Movie reviews.

Layer	Precision	Recall	F-Measure	Accuracy (%)	Time
**2**	0.96	0.96	0.96	95.61	7 m 23 s
3	0.84	0.86	0.85	85.05	9 m 24 s
